# Understanding the Radiobiology of Central Nervous System Diseases in the Golden Age of Radiosurgery—Does It Matter?

**DOI:** 10.3390/brainsci15060649

**Published:** 2025-06-17

**Authors:** Fred C. Lam, John Byun, Santosh Guru, Deyaldeen AbuReesh, Yusuke S. Hori, Elham Rahimy, Erqi Liu Pollom, Scott Soltys, David J. Park, Steven D. Chang

**Affiliations:** 1 Department of Neurosurgery, Stanford University School of Medicine, Stanford, CA 94304, USA; sg928@cam.ac.uk (S.G.); abureesh@stanford.edu (D.A.); yshori@stanford.edu (Y.S.H.); djpark@stanford.edu (D.J.P.); sdchang@stanford.edu (S.D.C.); 2 Department of Radiation Oncology, Stanford University School of Medicine, Stanford, CA 94305, USA; byunj@stanford.edu (J.B.); rahimy90@stanford.edu (E.R.); erqiliu@stanford.edu (E.L.P.); sgsoltys@stanford.edu (S.S.)

**Keywords:** stereotactic radiosurgery, Cyberknife, Gammaknife, proton beam radiation therapy, conventional radiotherapy, neurosurgery, neuro-oncology, arterial venous malformations, pituitary adenoma, functional neurosurgery, psychiatry, DNA damage response, primary brain tumors, metastatic brain tumors

## Abstract

Stereotactic radiosurgery (SRS) deploys image-guidance to deliver multiple beams of highly focused ionizing radiation to tightly conformed anatomical targets, leading to precise dosing of radiation-induced cellular injury and predictable biological responses that can be applied to treat a multitude of central nervous system (CNS) disorders. Herein we review the principles of CNS radiobiology, comparing differences between SRS and conventional radiation therapy. We then review the radiobiology of SRS as it pertains to the treatment of CNS tumors and vascular malformations and the emerging application of SRS for the treatment of functional and psychiatric neurological disorders. Finally, we look toward the future in combining SRS with other novel technologies to improve treatment outcomes for patients with CNS disorders.

## 1. Introduction and a Brief History of Radiosurgery

Stereotactic radiosurgery (SRS) delivers a high dose of focused ionizing radiation (IR) to a tightly defined target, leading to a desired local radiobiological response that can be usually achieved in one or two treatment sessions, while minimizing unintended radiation exposure to neighboring healthy tissues [1,2]. This distinct radiobiological advantage over conventional radiotherapy (cRT) has allowed for the wider use of SRS to treat central nervous system (CNS) disorders that were previously considered unsuitable for cRT, including the treatment of primary and secondary brain tumors, pituitary and other skull-based tumors, vascular malformations, and aberrant functional neurocircuitry [2,3,4,5,6].

The term SRS was coined by Swedish neurosurgeon Lars Leksell in 1951 [7]. However, the terms “stereotaxis” and “stereotactic” were first applied by British neurosurgeons Sir Victor Horsley and Robert H. Clarke to describe the Horsley–Clarke stereotactic frame in 1906, used to create electrolytic lesions in the deep cerebellum of monkeys [8,9]. Leksell designed his own stereotactic frame [10], drawing from modifications of the Horsley–Clarke frame and published his first paper using the term “stereotactic radiosurgery” to describe the precise pinpoint delivery of IR to the CNS [7], with the intention of using SRS to perform functional neurosurgery for epilepsy, facial pain, and neuropsychiatric disorders [11].

Over the next 15 years, Leksell’s physicist Börje Larsson experimented with a wide variety of sources of radiation, ranging from an X-ray dental tube to large and bulky orthovoltage equipment, to using non-Bragg peak cross-firing proton beams generated by the University of Uppsala’s 185-MeV research cyclotron [12], before eventually settling on a cobalt-60 source that would deliver high-energy gamma rays. They also worked tirelessly to study the radiobiology of delivering high-doses of IR to achieve an intended therapeutic effect within the brain [13], leading to the invention of the frame-based Gamma Knife (GK) radiosurgical device. The first iteration of the GK produced a large number of highly collimated beams of IR using “slit” lead collimators.

The first patient Leksell treated in 1967 using the GK had a craniopharyngioma, and the second patient he treated in 1968 had a pituitary adenoma [11]. Under Leksell’s mentorship, Tiit Rahn and Erik Backlund began treating patients with vestibular schwannomas using GK. By 1970, Ladislau Steiner alongside Leksell began to treat patients with arteriovenous malformations (AVMs) with SRS using biplanar stereotactic angiography to target the AVMs [14]. The “Golden Age of SRS” in America began when the University of Pittsburgh acquired a GK unit in 1987 under the leadership of L. Dade Lunsford, with more than 15,000 patients treated at that center to date [11,15]. Shortly thereafter, the University of Virginia established their GK SRS program under the leadership of Ladislau Steiner, who left Stockholm to take the helm, with more than 10,000 patients being treated there to date [11].

SRS treatments using linear accelerators (LINACs) began in the 1980s by Argentinian neurosurgeon Osvaldo Betti [16], in Italy by Francesco Colombo [17], and Juan Luis Barcia Salario in Spain [18]. Meanwhile, back in America, neurosurgeon Michael Apuzzo began performing image-guided stereotactic intracranial brachytherapy, incorporating frame-based techniques with computed tomography (CT) and magnetic resonance imaging (MRI) scans to implant iridium-192-containing catheters into glioma patients. Alongside his colleague in radiation oncology, Zbigniew Petrovitch, and Gary Luxton in radiation physics, they started the University of Southern California radiosurgery program, first using a linear accelerator, then moving to GK in 1989 [11].

Neurosurgeon William Friedman and physicist Frank Bova at the University of Florida, Gainsville, developed the first commercially sold frame-based radiosurgery platform, the Philips SRS200 (also known as the Linac Scalpel), with circular collimators ranging from 10 to 32 mm in 2.0 mm increments and a CT-based treatment planning system [19]. A circular collimator-based 6-MegaVolt (MV) LINAC system was sold briefly by Varians but was discontinued due to lack of demand. The germ of the idea of what developed into the CyberKnife arose from Adler’s exposure to stereotactic radiosurgery both in Stockholm and during his neurosurgery residency at MGH and the BWH, all of which eventually led to the idea of computationally correlating, in real time, live x-ray images with a previously acquired CT dataset. Thus spawned the birth of the Cyberknife robotic SRS platform, which is now owned by Accuray Inc. and used worldwide [20]. At Stanford Neurosurgery, where Adler is now Professor Emeritus, the Cyberknife SRS program, under the leadership of neurosurgeon Stephen Chang and radiation oncologist Scott Soltys, has treated over 10,000 patients with a variety of brain and spine disorders to date over a 27-year period [21]. Adler has continued to innovate with ZAP-X, a standalone, self-shielded 3 MV LINAC system that can deliver highly conformal intracranial SRS treatments [22].

Despite the theme of this review being centered around SRS, it would be remiss if we did not touch upon the invention of proton beam therapy in 1946 by Dr. Robert Wilson, with its ability to deliver a high dose of radiation at the Bragg peak, with a very steep decline in the radiation dose behind the target volume, reducing exposure to adjacent normal tissues [23]. The first synchrocyclotron was built and used to treat a tumor patient at the University of California, Berkley, in 1954 [24]. This was followed by the first hospital-based proton therapy facility at the Loma Linda University Medical Center in 1990. There are now over 100 proton beam therapy centers worldwide, with more than 60 centers under construction. For a more comprehensive review on the radiobiology of proton beam therapy, we would like to refer our readers to a summary of an international expert panel publication [25], as well as a recent bibliometric review of over five decades of proton therapy literature [26].

**Figure 1:**
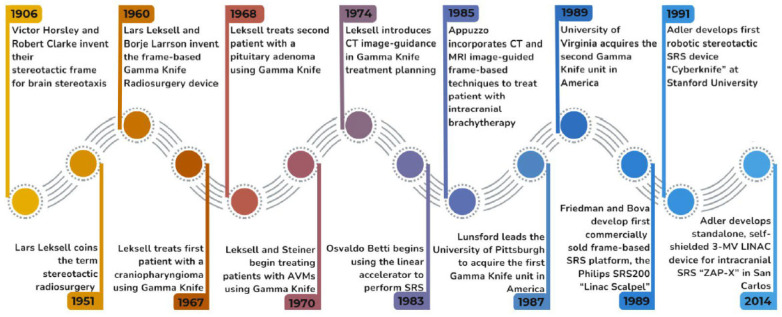
The History of Radiosurgery. A timeline of the key figures and events that helped shape the field of radiosurgery over that past century.

A timeline of the history of radiosurgery is provided in Figure 1. The increasingly widespread use of SRS for CNS diseases behooves neurosurgeons, radiation oncologists, neuro-oncologists, neurologists, and psychiatrists to have a firm understanding of the cellular responses to focused radiation so as to know when to appropriately offer this effective treatment modality to their patients. This review serves as a primer for understanding the radiobiology of radiosurgery and its effects on the CNS and offers a view at what is in store in the future for this exciting technology.

## 2. Principles of Radiobiology

Radiobiology is the study of the biologic effects of radiation on its intended target and surrounding healthy tissues [27]. When radiation is delivered to cells, it penetrates to the nucleus and causes DNA damage. The damage is primarily caused by reactive oxygen species (ROS) and free radicals that are generated by the interaction of photons with water. The ROS intercalate with DNA and cause either single-strand (SSBs) or double-strand breaks (DSBs). The prompt repair of DNA breaks is crucial in maintaining genome stability, which is essential for cellular survival [28,29,30].

**Figure 2:**
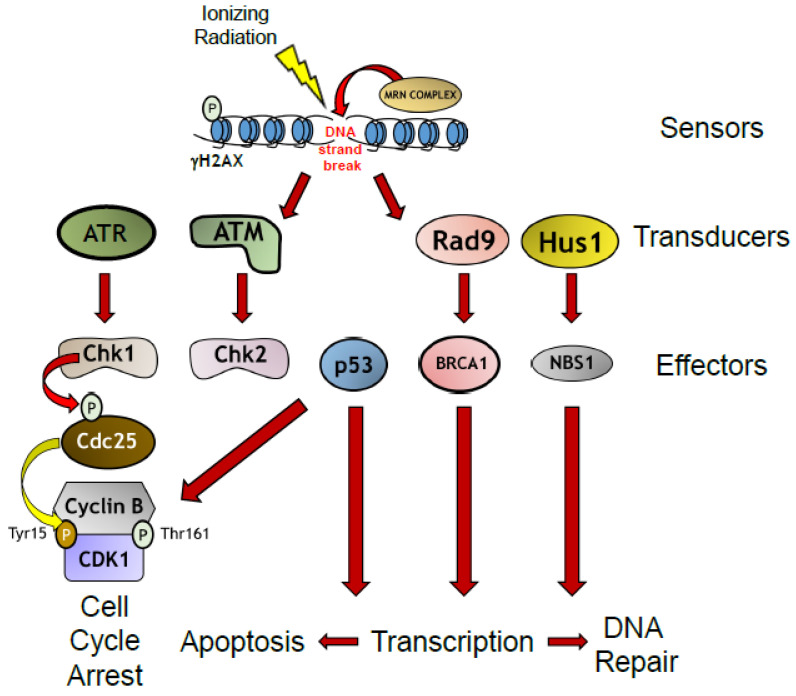
The DNA Damage Response in Response to Ionizing Radiation. Formation of radiation-induced double strand DNA breaks leads to recruitment of sensor proteins to break sites. Sensor protein complexes recruit transducer proteins which leads to phosphorylation of effector proteins that lead to upregulation of transcription of DNA repair proteins, or trigger cell cycle arrest, or apoptosis if the damage is irreparable.

The repair of damaged DNA is carried out by the DNA damage response (DDR), an evolutionarily conserved signaling cascade of protein kinases that senses DNA breaks, recruits protein complexes which can further amplify and transduce the damage signal, and leads to activation of effector protein complexes, which can do one of three things: (1) trigger G_1_/M cell cycle arrest to allow for break repair; (2) cause apoptosis because the damage is too extensive and the cell autodestructs to prevent passing on a damaged genetic code to progenitor cells; or (3) trigger cellular senescence (Figure 2) [31]. Not surprisingly, mutations in several DDR proteins cause debilitating neurological conditions such as ataxia telangiectasia and cancer syndromes such as hereditary breast and ovarian cancer [28,29]. As the field of DNA damage research is too vast for this review to cover, we refer our readers to several key reviews for further reference [28,32].

Understanding the physics of radiation in achieving a therapeutic radiobiological response in target tissues while protecting neighboring tissues allows for safe and effective delivery of radiation therapy. This involves understanding the physics of the linear energy transfer (LET) that occurs when a beam of IR traverses through tissues. The International Commission on Radiological Units and Measurements (ICRU) defines the LET as follows:

*“LET of charged particles in a medium is the quotient dE/dl, where dE is the average energy locally imparted to the medium by a charged particle of specified energy in traversing a distance of dl”.*

**Figure 3:**
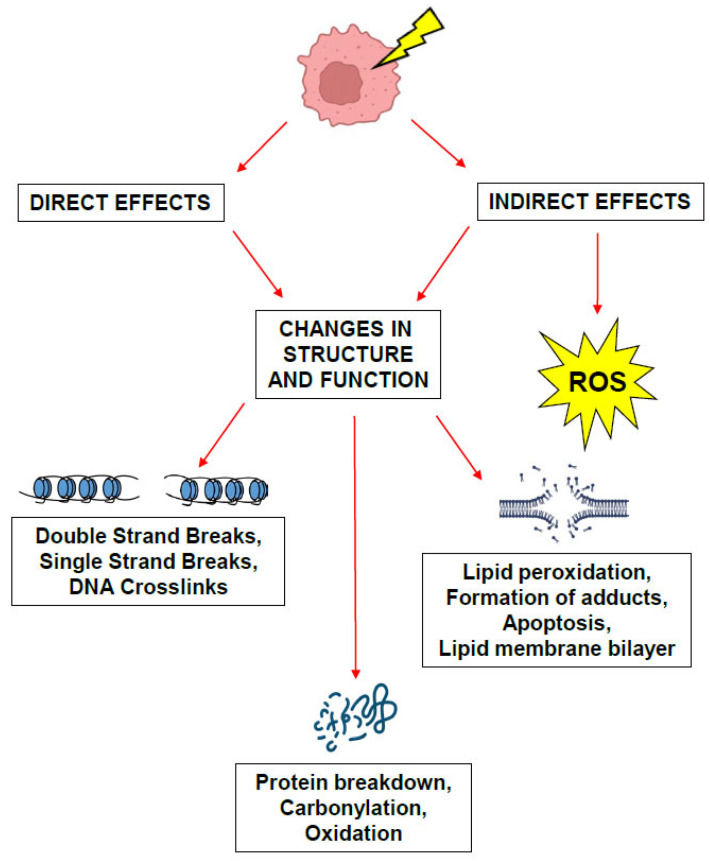
Mechanisms of biological damage caused by ionizing radiation. Direct and indirect effects of damage caused by ionizing radiation.

The LET is obtained by dividing the particle track into equal energy increments and averaging the track length over which the energy is deposited and is generally expressed in keV/µm [27,30,33]. X-rays and gamma rays are considered low-LET sources of radiation while protons, heavy charged particles, and neutrons are considered high-LET sources. The transfer of this energy leads to tissue damage through both direct and indirect mechanisms, whereby the ionizing energy directly damages the DNA of targeted cells and causes indirect damage by interacting with other molecules and atoms (namely water molecules as cells are composed of 80% water) in the cellular milieu to produce free radicals and ROS that can then diffuse throughout the cell to disrupt chemical bonds and produce chemical changes that lead to biological injury (Figure 3). High-LET particles such as protons and neutrons are thought to act through direct mechanisms of injury while low-LET particles such as X-rays are thought to exert their biological effects through indirect mechanisms of action [30,34]. The effects of this damage can cause effects which are either (1) acute and lethal to cells causing cell death; (2) sublethal, which can be repaired in hours unless further sublethal doses are administered, which eventually leads to lethality; or (3) potentially lethal, which may be overcome by repair mechanisms when the cells are in cell cycle arrest. This consequently can lead to lifetime somatic effects such as radiation-induced cancers and sterility, or genetic effects when germ cells are exposed, causing radiation-induced germline mutations that can be teratogenic to future progeny [35]. Extensive research over the past century has led to the establishment of the foundations of the radiobiology principles underlying the early success of conventional radiation therapy and more recently, the success of radiosurgery, which will be reviewed in the sections below.

In order to minimize the radiation dosage to non-target tissues, and with the goal of maximizing the dose to the target, radiation modalities utilizing different particles with differential LETs have been explored. Particle-based therapies, including protons, neutrons (neutron-beam and boron capture), alpha particles (helium nuclei), carbon ions, and other heavy particles like pi-mesons, suggest dosimetric advantages, achieving more favorable, localized dose distributions. Their mechanisms of cellular damage are under investigation, but in general are associated with increased double-stranded DNA breaks, particularly clustered DNA lesions that are less readily repaired through cellular repair mechanisms compared to photons. This increased complexity in DNA damage caused by proton or carbon ion therapy may elevate the probability of lethal chromosomal aberrations and subsequent cell death from mitotic catastrophe. Interestingly, studies have shown proton radiosurgery to achieve comparable local control rates of intracranial metastatic disease (approximately 90% at one year) to photon radiosurgery, illustrative of a comparable radiobiological efficacy despite different LET values. Carbon ion radiosurgery may be able to overcome tumor resistance, seen in hypoxic tumors or tumor subpopulations refractory to traditional photon-based therapies. Higher LET and the corresponding clustered damage induced by carbon ions render conventional DNA repair mechanisms less effective, thereby improving efficacy against radiation-resistant tumor phenotypes, including multiresistant tumor clones.

Relative biological effectiveness (RBE) is used to assess the amount of radiation-induced biological damage imparted to tissues with increasing doses of LET. RBE compares the prescribed dose to a standard dose of radiation to produce the same biological effect [35]. Traditionally, this standard was defined at 250 keV of X-rays, but it is more common now to use 1 MeV of photons from a cobalt-60 (^60^Co) source (i.e., an RBE of 1 is when comparing ^60^Co with itself) [30]. The RBE varies with the type of radiation, type of cell or tissue being targeted, dose rate, and fractionation scheme. Increasing the RBE alone does not offer any therapeutic advantage unless it creates a differential between the RBE of normal tissue and tumor tissue that leads to increased tumor cell killing and an increased therapeutic ratio across fractions; therefore, it is imperative to understand the biological effects of radiation dosing and fractionation.

As radiation may induce DNA damage in an oxygen-dependent manner, the oxygen enhancement ratio (OER) describes how tissue oxygenation can influence the response of cells to ionizing radiation. OER is defined as the ratio of radiation dose required to produce a specified biological effect under hypoxic (low oxygen) conditions relative to normoxic (normal oxygen) conditions [36]. Typically, OER values range between approximately 2.5 to 3.0 for X-rays or gamma rays commonly used in stereotactic radiosurgery (SRS), indicating that hypoxic tumor cells require around 2.5 to 3 times higher radiation doses to achieve equivalent cytotoxic effects compared to fully oxygenated cells [37]. In the laboratory, the surviving fraction of cultured Chinese hamster cells exposed to varying oxygen concentrations ranging from 0.0075 mm Hg O_2_ to ambient air, and radiation dosages ranging from 10 to 50 Gy, demonstrated significantly different surviving populations. A significant radiosensitivity increase occurs from approximately 0 to 30 mm Hg O_2_, which is approximately 0.5% O_2_, and then plateaus at 760 mm Hg at 37 °C. The presence or absence of adequate oxygen within tissues can profoundly influence clinical responses to radiotherapy and radiosurgery due to oxygen’s critical role in mediating radiation-induced cellular injury. Nonetheless, radiosurgery utilizes doses of radiation that are likely to overcome a low OER, even in hypoxic or necrotic tumors.

### 2.1. The Rs of Radiobiology in Conventional Radiotherapy

In the early 1970s, Withers introduced the “four R’s of radiotherapy” to describe the differential effects of dose fractionation on normal tissues compared to tumor tissues [38]. These involve the following: (1) *Repair* of sublethal injury in normal and tumor tissues; (2) *Reoxygenation* of the tumor; (3) *Redistribution* of tumor tissues through the cell cycle; and (4) *Regeneration* of surviving normal and malignant cells between dose fractions. Steel and colleagues later established the intrinsic radiosensitivity of cells as the fifth “R” [39] (Table 1). The complex interplay between these principles to optimize the redistribution of tumor cells into mitosis, where they are more sensitive to radiation and allow for reoxygenation of sublethally damaged populations of cells to further generate IR-induced ROS, can be leveraged across treatment fractions to maximize total tumor cell killing while minimizing normal tissue toxicities over the prescribed fractions of treatments [40]. While Steel’s fifth R of intrinsic radiosensitivity does not directly influence fractionation effects [39], factors that can lead to decreased radiosensitivity throughout a multifractionated treatment schedule include (1) the synchronization of cells into the more radioresistant late-S phase of the cell cycle; (2) creating a hypoxic tissue environment that prevents reoxygenation; and (3) the addition of chemical ROS scavengers [35].

The effectiveness of conventional RT is based on the linear quadratic (LQ) model of tumor cell survival following a single exposure to IR (this model is adapted from Berk) [27]:

SF = exp − (αD + βD^2^)

where for a single fraction

- SF = survival fraction after RT treatment;
- α = lethal lesions due to single-strand DNA breaks (single hit);
- D = delivered dose;
- β = lethal lesions due to double-strand DNA breaks (double hit).

This can also include correction for the time over which the treatment is given with the Lea–Catheside time factor (G), which can be introduced into the quadratic variable (GβD^2^).

This equation can then be used to calculate the biologically effective dose (BED), which is the dose needed to kill a fixed fraction of cells, and its derivative, the equivalent dose in 2 gray (Gy) fractions (EQD2), in which the standard international unit Gy is the mean energy imparted to and absorbed by a material. The BED is then calculated as

BED = D × (1 + [d/(α/β)])

and the EQ2 is calculated as

EQ2 = D × ([d + (α/β)/[2 Gy + (α/β)])

where

- D = total dose;
- d = dose per fraction;
- α/β = the alpha–beta ratio, based on the variables in the model above.

For aggressively growing tumors, the α/β ratio is ~10, and for slow growing tumors and normal tissues, the ratio is ~3 [27]. Normal tissues that have an early response to radiation have a high α/β ratio; normal tissues with a late response have a low α/β ratio [33].

The LQ model can be successfully applied to both in vitro and in vivo survival data at fractionated doses up to ~10 Gy [41] and may even be applicable to some tumors with single-fraction treatment at ~20 Gy [42]. However, to achieve an equivalent BED using higher doses of radiation would require either the straightening of the LQ curve beyond an arbitrary threshold dose or assuming higher α/β ratios for tumors [43]. This forced fit of the LQ model to accommodate higher doses of radiation may be achievable with in vitro survival data, but it would be limited in the clinical scenario across all tumor types. Secondly, the kinetics of DNA damage repair may plateau at higher doses of radiation, where the rate and extent of DSB repair is similar in cells after exposure to 1 Gy or 80 Gy of radiation, measured in vitro [44]. Thirdly, the LQ model has not been sufficiently applied to small target volumes (<2 cm in diameter). Therein lies the need to understand the mechanisms of biological effectiveness in high dose per fraction radiosurgery beyond the traditional 4 Rs.

### 2.2. The Radiobiology of Stereotactic Radiosurgery

Wither’s four Rs can still be applied broadly to SRS but given the limited numbers of fractions that can be used for SRS, and particularly in single-fraction treatments, only repair and radiosensitivity have significant effects. The mechanisms of tumor cell killing at higher doses of radiation are partly due to tumor endothelial cell apoptosis leading to indirect tumor cell death [45]. Secondly, the linear quadratic model may not be sufficient because it does not account for cell death due to effects on tissue stroma, vascular damage, or tissue hypoxia [46]. The G correction factor allows for clinically relevant doses of up to 10 Gy to 18 Gy per fraction [42]. Adjusting for these conditions, a fractionated SRS treatment can then be compared to a conventional RT regimen. For ease of reference, there are now published tables of equivalent doses for various α/β ratios and numbers of fractions for equivalent BED that are used in SRS treatment planning [47], as well EQD2 and BED online calculators.

In 2019, Boustani and colleagues proposed the reactivation of the antitumor response as the sixth R of radiobiology, in which radiation-induced DNA damage triggers the innate immune system, including the activation of pattern recognition receptors and interferon signaling [48,49]. Activation of the DNA damage-sensing kinase ATM by dsDNA breaks can activate the innate immune system through the upregulation of pattern recognition receptors (PPRs) such as Toll-like receptors, NOD-like receptors, and C-type lectin receptors, as well as the activation of the interferon signaling pathway [49]. The release of DNA into the cytosol is known to act as a danger signal through the cyclic GMP-AMP synthase (cGAS) to upregulate the adapter protein stimulator of interferon genes (STING) and the downstream activation of type I interferons [50,51], proinflammatory cytokines, and the recruitment of antitumor dendritic cells [52,53]. Preclinical experiments have shown that the release of cytosolic DNA peaks at 12 Gy per fraction then decreases, suggesting a mechanism of acquired radioresistance. This perhaps accounts for the loss of the abscopal effect at higher doses of radiation (20–30 Gy) and the increased infiltration of protumorigenic macrophages [54] compared to lower doses at repeated fractions (8 Gy × 3 fractions) [55].

A mathematical model showed that a conventional RT regimen of 1.8–2 Gy per fraction over the course of weeks leads to an immunosuppressive effect, correlating with treatment-related lymphopenia [56], reduced levels of IL-1β, and increased TGF-β, leading to an immunosuppressive tumor microenvironment (TME) [57,58]. On the other hand, normofractionated RT can have positive effects on the TME by normalizing the tumor vasculature and facilitating immune cell migration or upregulating antitumor macrophages [59,60].

A seminal study in irradiated mice lacking functional T cells showed a dampened tumor response to radiation [61], leading to a deeper understanding of radiation-induced antitumor immunity [62,63]. We now know that IR can lead to immunogenic cell death, leading to the activation of dendritic cells, effector T cell priming, the recruitment of cytotoxic CD8 and Th1 cells [64], and the upregulation of cell adhesion molecules such as intracellular adhesion molecule (ICAM)-1 and vascular cell adhesion molecule (VACM)-1, leading to lymphocyte adhesion to the tumor vascular endothelium [65]. Radiation also increases the expression of NKG2D receptor stress ligands on tumor cells, activating tumor cell clearance by natural killer (NK) cells [66]. This has led to the hypothesis that the irradiated tumor microenvironment (TME) becomes an in situ vaccine [48]. The phenomenon of the abscopal effect, the regression of metastatic tumors distant from the radiated treatment field, is evident of this induced vaccine state [67]. The abscopal effect will be discussed at a later point in this review.

**Figure 4:**
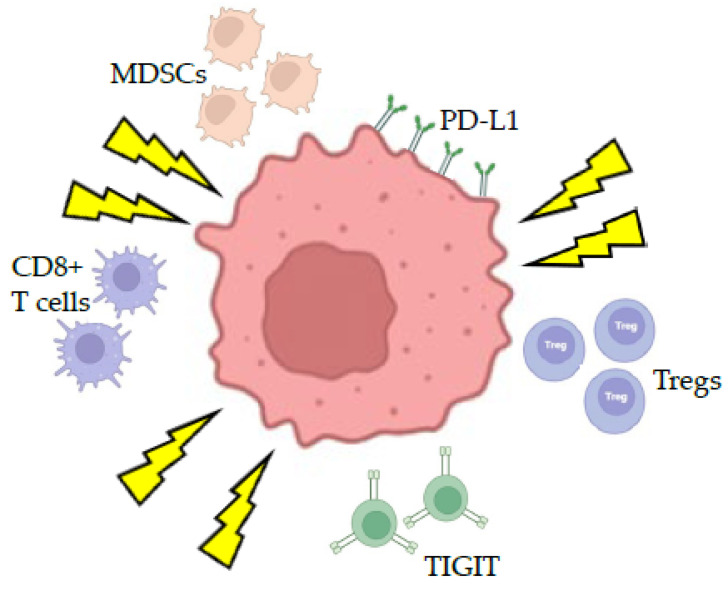
Response of the tumor immune microenvironment to ionizing radiation. Ionizing radiation can lead to immunogenic cell death leading to activation of effector CD8+ T cells, regulatory T cells, T cell immunoreceptor with Ig and ITIM domains (TIGIT). Radiation also increases the expression of the Programmed Death Ligand 1 (PD-L1) on the tumor surface.

Conversely, radiation can lead to an immunosuppressed state with increased infiltration of regulatory T cells (Tregs), tumor-infiltrating myeloid-derived suppressor cells (MDSCs) [68], and tumor-tolerant M2 macrophages [69]. Radiation can also increase the expression of programmed death ligand 1 (PD-L1) on the tumor surface and the upregulation of the T cell immunoreceptor with Ig and ITIM domains (TIGIT) [70], a co-inhibitory receptor expressed on CD8+ T cells, NK cells, Tregs, and T follicular helper cells (Figure 4). Thus, the ability to manipulate the immunostimulatory and immunosuppressive effects of radiation through fractionated treatment schedules may influence the immunogenic effects of radiosurgery.

Preclinical data has shown that doses greater than 12 Gy per fraction reduces the tumor immunogenicity and the abscopal effect [55], while a single 20–30 Gy dose of radiation to TSA breast and MCA38 colorectal tumors in mice reduced tumor immunogenicity and the loss of the abscopal effect compared to a smaller dose of 8 Gy given over three fractions [55]. High doses of radiation > 10 Gy have been shown to recruit protumorigenic macrophages [54], cause significant vascular damage and endothelial cell death, and decrease the recruitment of tumor effector T cells [45]. Mathematical models suggest that doses of 10–13 Gy of radiation per fraction maximizes antitumor immunity [71], while another study compared three fractionated protocols with the same BED: (1) 16.4 Gy × 1 fraction; (2) 8 Gy × 3 fractions; and (3) 2 Gy in 18 fractions. It was found that the 8 Gy × 3 fractions and 16.4 Gy × 1 fraction treatments upregulated the recruitment of Tregs and CD8+ T cells, while the 2 Gy × 18 fraction plan increased the recruitment of myeloid cells [72]. However, the observation that the proportion of tumor cell survival in orthotopic mouse models of cancer at single doses > 20 Gy can be predicted using the LQ survival curve at low doses (<10 Gy) [73] argues against the hypothesis that enhanced tumor killing is simply caused by endothelial damage, vascular collapse, or immunogenicity.

Fractionation was introduced, in part, to overcome the relative radioresistance of the hypoxic tumor environment [74]. Studies have shown that oxygen can diffuse approximately 150 µm from the capillary end artery to tumor surfaces, as such when tumors are less than 160 µm in diameter, there is no necrotic center. Tumors that grow beyond 200 µm have a center of necrotic cells and varying degrees of hypoxic cells in the outer layers [75]. Fractionation allows for the outer most layers of tumor cells to be removed and the reoxygenation of hypoxic cells to become more sensitive to subsequent fractions of treatment.

## 3. Biological Principles of Radiosurgery in the Central Nervous System

The intersecting beams of ionizing radiation in stereotactic radiosurgical treatment provides precisely targeted therapeutic doses with maximal conformality to specific CNS targets. This conformal targeting constructs a maximal radiation dose gradient to realize a minimum radiation dose to the designed target while preserving adjacent critical structures. Ongoing technological improvements have allowed progressively improved radiation dosing and initiated wider applications in neuro-oncological, vascular, and functional disorders. Ionizing radiation can generate cellular injury, particularly through DNA double-strand breaks (DSBs), apoptosis induction, mitotic catastrophe, and senescence. The resulting biological consequences are widespread, including differential acute and chronic CNS tissue responses, cell cycle sensitivity, vascular and cranial nerve responses, radiation tolerance limits, postoperative outcomes, and particularly late toxicities.

### 3.1. Acute Cellular Responses to CNS Irradiation (Days to Weeks)

A hallmark of acute CNS radiation exposure involves reactive changes within glial and endothelial cells, which is believed to occur primarily to cellular DNA. Radiation-induced DSBs activate rapid cellular responses, including the phosphorylation of histone protein γ-H2AX, the activation of repair mechanisms guided via ATM kinase signaling, and the subsequent recruitment and coordination of DNA repair machinery. Two distinct major, canonical repair pathways include nonhomologous end joining (NHEJ) and homologous recombination (HR). Within NHEJ, DNA ligase IV, DNA-PKcs, Ku70, Ku80, XRCC4, and XLF interact to efficiently repair damage but can be error prone, leading to cellular dysfunction. Within HR, BRCA2, Rad51, Rad52, Rad54, and RPA all interact in the late S/G2 cell cycle to promote slower but error-free, using sister chromatid template-based repair. There are many other important molecules in DNA repair from radiation damage, including ATM, MRE11, NBS1, and RAD50; notably, ATM is the protein mutated in ataxia telangiectasia, a disease characterized by extreme human radiosensitivity. Published reports show conflicting data for patients with ATM mutations regarding whether they should be receiving any radiation therapy. Even more limited data is available for radiosurgery, although a small case series notes no additional short-term radiation toxicity [76].

Acute radiation-induced DNA damage is diverse and accordingly has varied cellular repair timeframes as well. DNA damage leads to cell-level effects including apoptosis, autophagy, and senescence, for which dose-response symptomatology may be confounded by many treatment- or patient-specific factors. Numerous studies suggest transient disruption of the blood–brain barrier (BBB), interstitial edema formation, and acute neuroinflammation occur readily. Cytokines (IL-1β, IL-6, TNF-α), chemokines, and growth factors released by damaged endothelial cells and glia can create a transient inflammatory environment within hours to days post-SRS. In addition, astrocytes rapidly respond with reactive gliosis, transient glial proliferation, and morphological changes including demyelination and the appearance of perivascular infiltrate of lymphocytes. Neurons demonstrate some aspect of radioresistance in comparison to proliferating tumor cells, yet sufficiently high doses administered in radiosurgery can provoke acute neuronal damage and swelling in selectively irradiated regions.

General symptomatology in human models of acute radiation syndrome and exposure evolves over time and includes a possible stepwise development of neuromuscular symptoms. Larger doses of radiation are associated with the prodromal phase; patients treated with radiosurgery may develop easy fatiguability, apathy or listlessness, headache, and nausea in the initial few days to weeks after.

### 3.2. Normal Tissue Dose Constraints and Long-Term Toxicity of SRS in the Central Nervous System: Subacute and Late Cellular Responses to CNS Irradiation (Weeks to Months)

Subacute (weeks to months) and chronic (months to years) responses following radiosurgery reveal complex CNS biology. Radiation-induced DNA damage, transcription, and possible cell death likely trigger a series of interrelated biological cascades, including progressive fibrosis, chronic inflammation, endothelial remodeling, vessel sclerosis, and demyelination. An active area of ongoing study, CNS radiosurgery can trigger oligodendrocyte injury, initiating late demyelinating lesions. White matter pathology may present as periventricular and focal white matter changes on MRI, characterized by gliosis, axonal degeneration, decreased myelin integrity, and reduced functional neuronal connectivity, notably evident after higher-dose or repetitive radiation interventions. Additionally, radiation-induced late tissue reactions, commonly termed radiation necrosis, remain a major challenge. Necrosis reflects severe vascular damage, endothelial cellular apoptosis, fibrinoid necrosis, significant BBB breakdown, hypoxia, and progressive necrotizing inflammation. Damaged microvasculature and disrupted perfusion ultimately generate tissue ischemia and necrosis, typically appearing within 6–18 months post-radiation. Radiographically, late necrotic lesions can mimic tumor recurrence, complicating clinical differentiation [77,78,79].

Our current understanding of the dosimetric parameters to prevent IR-induced CNS toxicity is largely guided by published data using older planning techniques, many reporting toxicity following single-fraction SRS, without guidance on the potential benefits (if any) for fractionation. The use of high-resolution stereo MRI and CT imaging (i.e., ≤1 mm thick axial slices) in the simulation and contouring steps is imperative to offset the set-up uncertainty of ~1–2 mm limited by imaging voxel size and slice thickness that can increase the uncertainty in dose volume metrics, leading inadvertently to increased peripheral target doses being delivered to OAR. There is also a poor understanding of the regional variation of dose susceptibility in tissues and how comorbidities such as diabetes or cerebrovascular disease, prior exposure to radiation treatment, or neurosurgical procedures affect toxicity risk [80]. Nonetheless, this body of data provides a framework for our current understanding of normal tissue dose constraints in the CNS.

Historically, normal tissue complication probabilities (NTCPs) have been developed and published iteratively. Initially based on case reports, animal models, radiation biology modeling, and clinical outcome data, NTCPs vary based on publication date and aggregated data. Recently, several groups have independently published guidelines and tolerances for CNS SRS in the single- and multifraction setting. Milano and colleagues analyzed endpoints ranging from asymptomatic imaging lesions to clinically significant and symptomatic edema, noting that there was no universally accepted scale to grade radiation necrosis (https://pubmed.ncbi.nlm.nih.gov/32921513/, accessed on 30 May 2025).

The unique anatomy of the CNS with its highly radiosensitive structures and the inherently complex cellular organization of the brain makes SRS an ideal tool for treating a multiple of pathologies requiring the delivery of highly precise and conformal doses of IR. Furthermore, the post-mitotic nature of neurons makes the brain react differently to IR compared to other organs of the human body which continue to replicate and regenerate. The CNS is generally classified as a late responding tissue with an α/β ratio of ~2 [33]. Critical organs at risk (OARs) in the brain include the cranial nerves, cochlea, brainstem, and normal brain parenchyma; as such, care must be taken when conducting treatment planning around these OARs to ensure that they do not receive more than 50% of the maximal dose delivered to the target [80]. In this section, we review normal tissue dose constraints in the CNS and the complications of radiation exposure to OARs.

#### 3.2.1. Brain Parenchyma

Radionecrosis (RN) of the brain parenchyma is an irreversible late radiation-induced complication leading to the formation of a vascular lesion in the white matter in the irradiated field due to chronic inflammation of the brain parenchyma [81]. These lesions are characterized by hypocellular necrosis and fibrinous exudates. Radiation-induced dystrophic changes in the vasculature leads to the formation of telangiectasias, hyaline thickening of blood vessels, fibrinoid necrosis of small vessels causing ischemia, and intravascular thrombosis. The occurrence and severity correspond to the total dose given and the volume of brain exposed [82]. RN typically appears months to even years after radiation exposure (median time of 7–8 months post-SRS) [78]. A large series of 1650 patients with 2843 brain metastases treated with SRS reported an 8% incidence of RN with approximately 50% of cases being symptomatic [79]. The landmark Radiation Therapy Oncology Group (RTOG) 9005 SRS dose escalation study of recurrent brain metastases or gliomas reported an 8% and 11% 1- and 2-year incidence of RN, respectively [83]. Symptoms include motor or sensory deficits, changes in vision or speech, headaches, nausea, and somnolence, and can often mimic tumor recurrence [84]. A series reported by Korytko and colleagues found that occipital and temporal lesions and lesions in the pons/midbrain have a higher risk of developing RN, while frontal and temporal lesions have the lowest risk [85,86]. Lung adenocarcinoma, renal cell carcinoma, tumors with ALK rearrangement, HER2 amplification, and BRAF V600 mutation had increased risks of RN [78].

Risk factors for developing RN include (1) a conformality index (prescription isodose volume/tumor volume) > 2 and (2) a homogeneity index (maximum dose/prescribed dose) > 2. Larger tumors ≥ 1.6 cm in diameter had a ≥10% risk, while tumors < 1.5 cm had a ≤3% risk [87], with tumors > 10 cc having a >50% risk of developing RN [85]. The volume of normal brain receiving 10 Gy (V10 Gy) and 12 Gy (V12 Gy) with single-fraction SRS also correlates with RN risk, with rates increasing with increases in V12 [85,88]. Previous whole brain RT (WBRT) or SRS also increases the risk of developing RN, with the most important risk factor found to be prior SRS to the same lesion, up to a 20% 1 yr risk, a 4% risk of RN in the setting of prior WBRT, and 8% with concurrent WBRT. The risk of RN was only 3% with no prior RT. Evidence also suggests that the use of vascular epidermal growth factor tyrosine kinase inhibitors (TKIs) increases the risk of RN, as does vascular epidermal growth factor (VEGF) TKIs [89,90]. An SRS dose escalation study for primary and metastatic brain tumors conducted by the Radiation Therapy Oncology Group (RTOG), RTOG 90-05, reported the maximal tolerated marginal doses to be 24 Gy for <2.0 cm lesions, 18 Gy for 2.1–3.0 cm lesions, and 15 Gy for 3.1–4.0 cm lesions [83]. A study by Shehata and colleagues of 160 patients with 468 metastases ≤ 2 cm treated with SRS showed that peripheral doses < 20 Gy versus 20 Gy did not result in improved tumor control and appeared to be associated with a greater risk of grade 3–4 neurologic toxicity [91].

Treatments for symptomatic RN include corticosteroids, bevacizumab, laser interstitial thermal therapy (LITT), surgical resection, and/or hyperbaric oxygen [92]. Asymptomatic RN is generally observed with the possibility of combining oral pentoxifylline and vitamin E [93]. A recent systematic review on the management of symptomatic corticosteroid-refractory RN after SRS published by the International Stereotactic Radiosurgery Society outlined the evidence for each of these treatments and gave strong recommendations that carefully selected patients with corticosteroid-refractory RN not requiring surgical intervention can be treated medically with bevacizumab. The evidence for the role of LITT is poor, and there is not enough evidence to recommend hyperbaric oxygen [92].

#### 3.2.2. Brainstem

Studies have reported toxicity outcomes after SRS for brainstem metastases with a broad range of peripheral doses between 9 and 30 Gy, with median peripheral doses in the order of 15–20 Gy [94,95,96]. Given the eloquent location of these lesions, the patients tend to do poorly with a median survival of 5–11 months and may not have the opportunity to manifest late radiation toxicity. In a study from the University of Pittsburgh of 38 patients with benign tumors treated with SRS, the authors did not report any correlation between the marginal dose and adverse imaging findings or neurologic deficits [96]. Not all patients with adverse imaging findings developed neurologic deficits, and some patients developed deficits in the absence of adverse changes in imaging. The generally accepted dose constraint for the brainstem is 12 Gy based on studies that have shown that a brainstem maximum dose of 10–12 Gy is expected to result in minimal (<2%) risk of brainstem toxicity [80].

#### 3.2.3. Optic Nerve and Chiasm

Early data from the 1990s reported that maximal doses < 8 Gy to the optic nerve and chiasm resulted in low (0–1%) risks of optic neuropathy; however, these values predated the use of MRI combined with CT for contouring and treatment planning [97,98]. More recent studies support that a maximal dose of 10–12 Gy to the optic apparatus is well-tolerated with a low (0–3%) risk of symptomatic optic neuropathy. Recommendations from the Quantitative Analysis of Normal Tissue Effects in the Clinic (QUANTEC) publications, which offers a cooperative, in-depth review of the literature on normal tissue effects after radiation, suggest that the risk of optic neuropathy is minimal with maximal doses < 8 Gy, while the risk of symptomatic neuropathy increases to 10% with maximal doses of 12–15 Gy [99].

#### 3.2.4. Cranial Nerves of the Cavernous Sinus

Cranial nerves (CN) III (oculomotor), IV (abducens), V_1_ and V_2_ (branches of the trigeminal nerve), and VI (abducens) run through the cavernous sinus en route to innervate the extraocular muscles (CN III, IV, and VI), and sensation to the forehead and mid-portion of the face (CN V_1_ and V_2_, respectively). The maximal dose tolerance of these nerves is derived from the SRS/SRT treatment data of patients with cavernous sinus meningiomas or pituitary adenomas [100,101,102], which showed that SRS improved pre-existing CN III to VI neuropathies due to tumor compression in up to two-thirds of patients with cavernous sinus meningiomas [101,102,103]. There is no defined dose-response relationship for toxicity of the nerves within the cavernous sinus. The root entry zone of the trigeminal nerve as it exits the brainstem heading toward the cavernous sinus can tolerate up to 90 Gy, which is commonly the prescribed dose to treat trigeminal neuralgia.

#### 3.2.5. Vestibulococchlear Complex

The vestibulocochlear nerve (CN VIII) exits the brainstem and travels through the porous accousticus to innervate the cochlea and semicircular canals for hearing and balance, respectively. The maximum tolerated dose data for these structures comes from vestibular schwannomas (VSs) treated with SRS. The cochlea, nerve complex, and brainstem are considered OARs, and data from this patient cohort has shown that doses in excess of 12–13 Gy can lead to significant hearing loss [104,105]. The data from these can be difficult to interpret as hearing loss is a presenting symptom of VS, but a recent systematic review of over 4234 patients validated the 12–13 Gy dose constraint, independent of tumor size [106].

#### 3.2.6. Facial and Trigeminal Nerves

The facial nerve (CN VII) exits the brainstem in proximity to CN VIII and enters the porous accousticus as a CN VII and VIII nerve complex, then exits the base of the skull through the stylomastoid foramen to innervate the facial muscles. The trigeminal nerve (CN V) splits into three branches (V_1_, V_2_, and V_3_) as it exits the foramen ovale to innervate the face. Extrapolating again data from acoustic neuroma patients treated with SRS, doses in excess of 12–13 Gy are associated with significant risks of facial nerve palsy and trigeminal neuropathy [107,108].

#### 3.2.7. Blood Vessels

The current understanding of the radiation effects on blood vessels comes from the data on treating arteriovenous malformations (AVMs) using SRS. Direct exposure to high doses of radiation leads to endothelial cell injury, hyalinization, and the thickening of vessel walls [109]. Preclinical studies showed that a 25 Gy SRS dose inhibited Notch1 and 4 receptor signaling, upregulated apoptosis, and induced thrombosis in the nidus of rat models of AVM [110]. Clinically, a median prescription dose of 19 Gy to a median nidus volume of 5.7 cc leads to the cumulative obliteration of 44% of patients treated over a median time of 43 months [111]. Furthermore, SRS doses greater than 20–23 Gy to the cavernous sinus leads to carotid artery stenosis [112], thrombosis, and occlusions [113].

Understanding the normal tissue dose constraints of the CNS leads to safer delivery of high-dose SRS, recognizing that there will also be some level of radiation exposure to surrounding normal tissues. Continued improvements in imaging technology to achieve the higher resolution of neural structures and the increased sophistication of SRS planning software should help to decrease risks of inadvertent treatment toxicity.

## 4. Radiobiology of Radiosurgery for Brain Tumors

### 4.1. Benign Tumors

There are now decades of data supporting the efficacy of SRS for the treatment of benign brain tumors, including VS, meningiomas, and pituitary adenomas. Due to the inherently slow growing nature of benign intracranial tumors and the effectiveness of SRS in halting further tumor growth, patients rarely need to undergo subsequent surgical resection, leading to a paucity of data regarding the radiological effects of SRS on these types of benign tumors.

An elegant prospective series published by Alomari and colleagues from the Yale Gamma Knife Center in 2014 prospectively enrolled 1100 patients with both benign and malignant brain tumors, who received single-fraction SRS over an 8-year period with clinical and radiographic follow-up [114]. Patients were offered surgery if they developed etiologically ambiguous, gadolinium-enhancing lesions, if they became symptomatic, or if obtaining a tissue diagnosis was required for further patient management. Separate biopsies of the enhancing and non-enhancing regions of each tumor were obtained under stereotactic guidance along a single trajectory. Samples were then sent for histopathological analyses to ascertain differences in radiobiological response between the enhancing and non-enhancing regions. For WHO grade 1 meningiomas treated with SRS, enhancing regions revealed inflammation, demyelination, and cystic changes, while non-enhancing regions showed areas of coagulative necrosis, edema, vasculopathy, and cystic changes. These types of changes have also been reported for other benign tumors such as VS and pituitary adenomas [115]. Furthermore, it has been reported that there is a doubling of apoptotic cells within the first 48 h after SRS for VS and meningiomas [33].

#### 4.1.1. Vestibular Schwannomas

The treatment of xenograft mice with human vestibular schwannoma cells orthotopically transplanted into the subrenal capsule with 10, 20, or 40 Gy of SRS demonstrated cytotoxic changes at 20 and 40 Gy with associated vascular changes, hemosiderin deposition, and mural hyalinization [116]. These earlier preclinical experiments have guided the current understanding of the biological response of benign CNS tumors to SRS. Current treatment paradigms for VS have swung significantly in favor of SRS, with up to a 41% decrease in rates of surgical resection for these benign tumors [117,118]. The advent of more sophisticated stereotactic planning software using both high-resolution MRI and CT imaging has allowed for improved conformal dose planning and the use of more isocenters of radiation and smaller irradiation beams. This has allowed for reductions in the average dose to the tumor margin to 12–13 Gy with the 50% isodose line being used in 90% of patients, which can be further adjusted and tailored based on tumor volume, the baseline function of CN VIII, and patientsl’ presenting symptoms, significantly reducing comorbidities associated with the treatment of VS using SRS [105,118,119].

Hearing preservation by limiting the dose to the cochlea using beam blocking and the smallest collimators with a maximal tolerated dose of 4 Gy to the cochlear apparatus have significantly decreased hearing loss, especially in patients with intracanalicular VS [104,120]. While short term data reported ~60–85% preservation of hearing in patients who had serviceable hearing heading into their SRS treatments [121], longer-term results have reported 57% preservation at 5 years and only 24% at 10 years [122]. Furthermore, a study comparing the rate of hearing loss following SRS to the natural history of hearing loss for VS found an annual reduction of 3.77 dB per year following SRS compared to 5.39 dB per year before SRS. The rate of hearing loss following SRS was dependent on the cochlea receiving a <4 Gy dose [123]. Fractionated SRS does not appear to be superior to single-fraction SRS, likely indicative of the high sensitivity of cochlear hair cells to DNA damage caused by SRS [124].

#### 4.1.2. Meningiomas

Meningiomas are extra-axial lesions that are believed to grow from the arachnoid cap cells of the dura mater. Benign WHO Grade 1 meningiomas respond well to SRS, leading to >90% long-term control. A cohort study of 972 patients with 1045 meningiomas from the University of Pittsburgh reported a 93% control rate in patients with WHO Grade 1 meningiomas who received adjuvant SRS prior to resection, while primary SRS patients without a biopsy-confirmed histologic grading achieved a tumor control rate of 97% [125]. Control rates were proportionately lower in patients with WHO Grade 2 and 3 tumors who received adjuvant SRS (50% and 17%, respectively), with some studies showing a dose-response relationship for WHO Grade 2 and 3 tumors using tumor margin doses > 15 Gy. Despite previous reports of radiation-induced secondary tumors following the treatment of meningiomas with RT, this study did not endorse any cases of radiation-induced chondrosarcomas or gliomas [126,127].

The proven efficacy of upfront SRS for meningiomas has decreased the need to expose patients to surgical morbidity and mortality, particularly in the elderly population, especially when a Simpson Grade 1 curative resection (i.e., including a 1 cm margin of unenhancing dura) is not achievable. Postoperative SRS to the residual tumor is also now recommended rather than waiting for signs of tumor growth on surveillance imaging [103]. This is also true for small meningiomas in regions of the brain that are surgically difficult to access [115].

#### 4.1.3. Pituitary Adenomas

Nonfunctioning pituitary adenomas make up ~30% of all pituitary tumors. The goals of SRS are to control tumor growth, preserve normal pituitary function, and decompress the optic apparatus without compromising optic nerve function [128], and should be reserved for small adenomas (<3 cm in diameter) that are well-defined and away from the optic chiasm (≥3 mm) [129]. A study published by the University of Virginia using GK SRS reported a 92% tumor control rate with 25% presenting with endocrine dysfunction 2 years post-treatment [130]. The North American Gamma Knife Consortium published their pooled results of 512 patients with nonfunctioning pituitary adenomas, 479 of whom had undergone surgical resection and who were treated with a median dose of 16 Gy SRS to the tumor margin. The actuarial tumor control rates were 98%, 95%, 91%, and 85% at 3, 5, 8, and 10 years after treatment with SRS, respectively. Approximately 20% of patients had new or worsened hypopituitarism after SRS, which may be a side effect of radiation to the pituitary stalk and can present as late as 10 years following SRS treatment [131].

A series from the University of Pittsburgh that evaluated outcomes in patients with secreting pituitary tumors found that patients with acromegaly responded best to SRS with normalization of growth hormone secretion in > 70% of patients [132]. Patients with persistent Cushing’s disease after resection who received SRS to a mean marginal dose of 22 Gy achieved 98% tumor control and 70% remission of their Cushing’s disease at a mean 48-month follow-up [133], while 27% of patients with invasive prolactinomas had resolution of their endocrine dysfunction with 55% demonstrating improved endocrine function after SRS at 36 months median follow-up [134]. The recommended dose of SRS delivered to the adenoma ranges between 12 and 20 Gy in a single fraction with higher doses (16–20 Gy) reserved for functioning/secretory adenomas, keeping the maximum dose to the optic chiasm < 8 Gy [135,136].

Patients receiving SRS or SRT to the sella region have a 4% cumulative probability of developing radiation-induced secondary neoplasms, including gliomas, meningiomas, and sacromas [137,138]. A multicenter, retrospective study of 4292 patients with pituitary adenomas or craniopharyngiomas showed that prior SRS treatment was associated with an increased risk of developing a radiation-induced brain tumor with a rate ratio of 2.18 (95% CI, 1.31–3.62, *p* < 0.0001) and a cumulative probability of 4% compared to 2.1% at 20 years in patients who did not receive radiation [139]. A second study of 426 patients who received radiation treatments for their pituitary adenomas demonstrated a cumulative risk of 2.0% at 10 years and 2.4% at 20 years [140]. A systematic review of 180 cases of radiation-induced intracranial sarcomas reported that patients received an average dose of 51.4 ± 18.6 Gy to the brain with a latency period of developing sarcomas of 12.4 ± 8.6 years. Forty-nine of these cases (27.2%) were patients who received radiation for their pituitary adenomas [141]. The risk of developing radiation-induced secondary malignancies is reported to be much lower with SRS than with conventional EBRT, with an estimated cumulative incidence of 0.045% over 10 years, presumably due to the increased conformality of dose delivery to the tumor with the shielding of neighboring healthy tissues [142].

#### 4.1.4. Craniopharyngioma

Craniopharyngiomas are benign, neuro-epithelial tumors derived from the remnants of Rathke’s pouch or the hypophyseal duct. Due to its proximity to the pituitary, optic apparatus, hypothalamus, and cavernous sinus portion of the internal carotid arteries, the management of these tumors is a combination of surgical resection and radiation therapy [143,144]. Treatment of craniopharyngiomas with SRS alone is usually reserved for smaller (<3 cm in diameter) tumors that are ideally several millimeters away from the optic apparatus [145,146]. A mean marginal dose of ~12 Gy to the enhancing, relatively radiosensitive solid component of the tumor leads to tumor control rates at ~80% with one case series reporting a 90% control rate in solid tumors and a 58.6% control rate in mixed tumors [147]. Two large case series using GK SRS reported morbidity rates of 4% [147] and 6.1% [148] following treatment with SRS, and a mortality rate of 0.5% [147]. Common complications included vision loss and endocrine dysfunction (diabetes insipidus) [115].

### 4.2. Malignant Tumors

#### Glial Neoplasms

The collaboration between neurosurgeon Michael Apuzzo and radiation therapists Frederick George and Nisar Syed at the University of Southern California (USC) in the 1970s to establish a neurosurgical brachytherapy program for the treatment of gliomas highlights the rich history of using radiation as a modality to treat intrinsic malignant brain neoplasms. Apuzzo used the BRW stereotactic frame at USC alongside CT image-guidance to place catheters into gliomas to deliver iridium-192 brachytherapy [11]. The passion and curiosity that Apuzzo had for using radiation in the treatment of malignant tumors led to strong partnerships between radiation oncologists (Zbigniew Petrovitch) and radiation physicists (Gary Luxton) in the early inception of the radiosurgery programs at USC for the treatment of intracranial neoplasms [149].

Glioblastoma (GBM) is the most common and aggressive primary brain tumor in adults and accounts for approximately 15% of all brain tumors [150]. The current standard of care treatment includes a maximal safe surgical resection followed by conventional RT, and concomitant and adjuvant temozolomide affords patients a median survival of 15 months [151,152]. The disease inevitably recurs, leaving little options for salvage, including use of chemotherapies and SRS [153,154,155]. Molecular profiling of GBM tumors is now routinely performed for prognosticating treatment response and survival. GBM tumors with wild-type isocitrate dehydrogenase 1 and 2 (IDHwt) carries a worse prognosis than those harboring IDH1/2 mutations (IDHmut) [156], while the presence of O^6^-methylguanine-DNA methyltransferase (MGMT) methylation is associated with a better response to temozolomide (TMZ) chemotherapy [157]. Furthermore, MGMT promoter methylation combined with the ability to achieve a gross total resection of the enhancing disease in patients with IDHwt gliomas is associated with significantly favorable overall survival (OS) and progression-free survival (PFS) [158,159].

SRS has been used to treat recurrent GBM disease with some survival benefit and acceptable toxicity. A recent study by Bunevicius and colleagues assessed the safety and efficacy of SRS (median dose 15 Gy and median treatment volume 7.01 cm^3^) for patients with recurrent IDH-wt GBM in a retrospective observational international multi-institutional study [160]. All patients received surgery and TMZ, and 98% underwent fractionated RT. The median post-SRS PFS was 4 months. An SRS treatment volume > 5 cc was an independent predictor of shorter post-SRS OS. Radiation necrosis was diagnosed in 10% of patients and was managed conservatively. In a univariate Cox regression analysis, the only thing that predicted a longer PFS was an SRS dose > 14 Gy, while treatment volumes > 5 cm^3^ were associated with a shorter OS, and a higher Karnofsky Performance Score (KPS) was associated with longer post-SRS overall survival (OS).

It has been established that a smaller SRS treatment volume (< 14 cm^3^) is associated with better prognosis after SRS [161,162,163,164], and an earlier study reported that a higher prescribed dose > 15.5 Gy is associated with longer OS [165]. However, these studies were published before the era of the molecular biomarker testing of patient GBM samples. A recent study by Dono and colleagues in a cohort of 43 patients with IDHwt gliomas did not find any association between SRS dose, PFS, and OS [166]. MGMT promoter methylation is associated with a longer PFS and OS of newly diagnosed and recurrent GBM patients treated with SRS [89,167,168]. The presence of PTEN mutation is also associated with longer OS after salvaging SRS for IDHwt GBM [166].

IDH1 and IDH2 are metabolic enzymes that catalyze the oxidative decarboxylation of isocitrate to generate α-ketoglutarate (αKG) and carbon dioxide [169]. Hotspot mutations in *IDH1* and *IDH2* occur in various human cancers, including GBM, acute myeloid leukemia (AML), and cholangiocarcinoma. The cancer-associated mutation almost always occurs at distinct arginine residues in the enzyme active sites [170,171], leading to neomorphic enzyme activity that catalyzes the conversion of αKG to D-2-hydroxyglutarate (D2HG) and the accumulation of supraphysiologic levels of D2HG within cells [172]. D2HG inhibits aKG-dependent dioxygenases that are involved in the regulation of epigenetics and differentiation. In GBM and AML cells, D2HG accumulation causes epigenetic dysregulation that is thought to induce a DNA hypermethylation phenotype, which is clinically associated with the increased methylation of tumor DNA, leading to the glioma-associated CpG island hypermethylator phenotype [173,174]. D2HG-induced dysregulation of histone and DNA methylation inhibits normal cellular differentiation and promotes pathological self-renewal of stem-like progenitor cells, promoting a cellular state that lends itself to malignant transformation [169].

A recent in vitro study by Han and colleagues assessed the effects of 5 Gy of ionizing radiation on IDH1^R132H^ mutant U87MG and U251MG human glioma cell lines [175]. They evaluated the DNA damage response, cellular proliferation, migration, and invasion function and found that IDH1mut glioma cells have a higher radiosensitivity than wild-type cells but have decreased proliferation, migration, and invasion capabilities. They then analyzed IDH1 gene status and the survival data of 83 GBM patients who received radiotherapy at their institution between 2017 and 2020 and performed CGGA and TCGA analysis to find differential gene signatures between IDH1wt and IDH1mut GBM patients. Their results showed that IDH1mut GBM patients had statistically significant longer OS and PFS versus IDHwt GBM patients. Through their CGGA and TCGA analyses, they established a risk model that can predict the efficacy of radiotherapy for GBM patients. They identified a four gene radiotherapy-related signature including *ADD3*, *GRHPR*, *RHBDL1*, and *SLC9A9*. Patients in the high-risk group had worse OS compared to the low-risk group. High- and low-risk groups of patients receiving RT have significant survival differences, while patients who never received RT had no survival difference. OS was dependent on the high expression of these four genes [175]. A separate study reported that glioma cells expressing IDH1R132H mutant protein have reduced clonogenic ability after radiation compared with IDH1wt, suggesting that IDH1^R132H^ epigenetically reduces the expression of TIGAR, a regulator of intracellular redox homeostasis, leading to the inability to clear IR-induced ROS [176]. A third study showed that the IDH1 small-molecule inhibitor AGI-5198, currently being used in clinical trials to treat IDH1mut glioma patients, could reverse the DNA DSBs and cell death caused by IR [177]. These new findings point toward the potential to enhance the efficacy of SRS for the treatment of patients with IDH1mut GBM.

We recently published a phase I/II trial to determine the safety of delivering five fractions of SRS with 5 mm margins with concurrent TMZ in patients with newly diagnosed GBM irrespective of tumor molecular subtype [167]. Patients were enrolled in a 3 + 3 trial design where they received 5 days of SRS on four escalating dose levels of 25, 30, 35, and 40 Gy. Dose limiting toxicity (DLT) was defined using the presence of Common Terminology Criteria for Adverse Events (CTCAE) grades 3–5 acute or late CNS toxicity, including adverse radiation effect (ARE), the imaging correlate of radiation necrosis. Thirty patients were enrolled with a median target volume of 60 cm^3^. DLT occurred in two patients: one, for cerebral edema and disease progression at 3 weeks following SRS and TMZ treatment (CTCAE grade 4, dose 40 Gy); and the second patient died from postoperative complications 1.5 weeks after SRS (CTCAE grade 5, dose 40 Gy). With a median follow-up of 13.8 months, median PFS was 8.2 months, median OS was 14.8 months, and the median survival of patients with MGMT hypermethylation was 19.9 months vs. 11.7 months with no/unknown methylation status. Median OS was 27.2 months if no ARE occurred vs. 11.7 months if late ARE occurred. MTD was determined at 40 Gy in 5 fractions and ARE was limited to grades 1–2 and did not affect survival. This study highlights the possibility of using SRS to prime the TME to achieve an immunostimulatory effect, which may enhance the efficacy of TMZ and/or immunotherapy for GBM patients and provide patients with less burdensome radiotherapy treatment options compared to the standard of care 60 Gy in 30 fractions of radiotherapy [151].

SRS has also recently been applied with good effect for the reirradiation of GBM upon recurrence. Mayer and Sminia published a study in 2008 using clinical brain reirradiation cases performed at their institution from 1996 to 2006 to assess the presence of radiation necrosis follow reirradiation and found that the normalized total doses (NTD_cumulative_) to healthy brain in conventional reirradiation series (NTD_cumulative_ of 81.6–101.9 Gy) were lower than the NTD_cumulative_ values for FSRT- or LINAC-based SRS case series [178]. They did not find a correlation between the time from the initial RT treatment to reirradiation and the incidence of radiation necrosis. ESTRO and EANO recently put out guidelines for reirradiation of GBM and recommend hypofractionated SRT (36 Gy in 2 Gy fractions) for moderate-to-large lesions, or single-fraction SRS for smaller lesions (16–24 Gy with prescription to the 50–80% isodose line, GTV size up to 3 cm diameter) [179]. The estimated risks of symptomatic brain necrosis following single-fraction SRS stratified to volumes of normal brain receiving 12 Gy (V_12Gy_) of 5 cc, 10 cc, or >15 cc are approximately 10%, 15%, and 20%, respectively [84,180]. Prior SRS to the same treatment area increases the risks of brain toxicity by ~5-fold with a crude rate of ARE of 14% and a 1-year cumulative incidence of ARE of 20% [87].

Lastly, the role of preoperative SRS to GBM is being explored to help improve outcomes. The purported advantages of preoperative SRS include the ability to achieve increased dose delivery, intact tissues have higher oxygen concentrations, allowing for more effective IR-induced DNA damage, and the activation of the immune TME in response to radiation [181]. Furthermore, the potential to leverage the radiobiological effect of SRS to prime the immune TME (the sixth R of radiobiology) may improve a GBM tumor’s response to immunotherapy. Preclinical studies have shown that IR regulates the expression of HLA class I antigens on the surface of human glioma cell lines [182] and that the treatment of a syngeneic murine model of glioma with IR with peripheral vaccination leads to long-term survival [183]. Zeng and colleagues published a preclinical study in a syngeneic intracranial murine model of glioma and found that the treatment of mice with SRS to a dose of 10 Gy with anti-PD-1 therapy significantly enhanced survival above and beyond anti-PD-1 or SRS alone [184]. The NeoGlioma Study (NCT05030298) to evaluate the effectiveness of preoperative SRS in high-grade glioma prescribes a PTV margin of 3 mm due to the use of LINAC SRS. The GTV is contoured using the T1 contrast sequence to encompass all of the enhancing part of the tumor. A CTV margin is not applied in the preoperative setting, but once the patient undergoes surgical resection, CTV is used to address subclinical diffuse infiltrative disease. The optimal timing for surgery after the initial dose of SRS to allow enough time for immune priming of the radiated tumor bed is still under investigation. In the NeoGlioma study, surgical resection is recommended within 14 days after SRS. This trial highlights the potential for further research into understanding the radiobiology of SRS in glioma cells which can lead to much needed novel therapeutics to improve patient survival.

### 4.3. Brain Metastases

Volker Sturm and colleagues were the first to report the use of LINAC radiosurgery to treat a patient with a deep-seated brain metastasis in 1987 at the German Cancer Research Center in Heidelberg [185]. This was followed by a report from Stockholm by Christer Lindquist in 1989 using Gamma Knife to treat a resected, recurrent renal cell carcinoma brain metastasis [149] and a case series in 1990 from Jay Loeffler and Eben Alexandra at the Brigham and Women’s Hospital in Boston detailing the treatment of patients with recurrent brain metastases using LINAC radiosurgery [186].

The well-circumscribed nature of brain metastases lends itself to being an ideal target for the highly conformal nature of SRS, allowing for a steep dose fall-off to minimize toxicity to uninvolved, neighboring brain tissue. SRS is now frequently used as a single modality for oligometastatic disease alone or in conjunction with systemic chemotherapy, targeted therapy, or immunotherapy [187,188,189,190]. Furthermore, the ability of SRS to mitigate the subacute and chronic adverse effects of WBRT, including hair loss, fatigue, and cognitive decline, has led to SRS being the preferred radiation modality for the treatment of brain metastases [191,192,193,194,195]. Furthermore, the convenience of outpatient SRS treatments in tandem with systemic therapies for effective intracranial disease control has allowed for SRS to become a powerful adjunct to the surgical resection of patients with asymptomatic brain metastases [115].

Current guidelines from the American Society of Clinical Oncology (ASCO), Society for Neuro-Oncology (SNO), and American Society for Therapeutic Radiology and Oncology (ASTRO) recommend single-fraction SRS for patients with metastases measuring 3 cm in diameter, and fractionated SRS is conditionally recommended for lesions ≥3 to 5 cm in diameter [196,197,198]. A study from MD Anderson looked at 1- and 2-year local control rates in patients with brain metastases treated with SRS and reported significantly lower rates of control (56% and 24%, respectively) in patients with lesions that were greater than 1 cm in diameter (0.5 cm^3^) compared to lesions less than 1 cm in diameter (86% and 78%, respectively) [199]. Tumor histology subtype, tumor location (eloquent versus non-eloquent cortex), and effectiveness of primary site disease control likely all contribute to the intracranial response to SRS [200]. Renal cell carcinoma, colorectal cancer, sarcoma, and BRAF positive melanoma are considered relatively radioresistant [201,202]. A recent single-institutional retrospective study of 1095 patients with a total of 1733 treatment-naïve brain metastases < 3 cm who received frame-based SRS over a 25-year period identified variables that influenced time to local treatment failure (TTF) and local control rates (LCRs) [203]. Multivariate analysis identified that age, year of SRS, tumor size, and primary tumor histology significantly influenced TTF, with melanoma having a significantly shorter TTF relative to non-small cell lung carcinoma (NSCLC) and renal cell carcinoma (RCC). NSCLC and RCC had higher 2 yr LCRs compared to melanoma and breast cancer. Melanoma and breast cancers had lower 2 year LCRs. This study stresses the importance of not relying on lesion size as the sole indicator that guides the SRS treatment of brain metastases and the need for further understanding of the radiobiology of brain metastases of different tumor histologies.

A recent single-institutional study by Upadhyay and colleagues reported one of the largest cohorts of patients who received SRS for ≥15 brain metastases in 1 to 5 fractions over an 8-year period and concluded that SRS was safe and provided comparable cognitive and survival benefits to studies using WBRT in this population with oligometastasis [204]. Of the 118 patients treated with LINAC-based SRS, the median number of lesions treated per patient per course was 20 (range, 15–94), with most patients (81.5%) receiving 24 Gy in 3 fractions. The rates of any grade of RN and grade ≥3 RN were 15.3% and 3.2%, respectively. Prior treatment with RT had a negative effect on median survival, and 1-year freedom from neurologic death, leptomeningeal disease, and salvage WBRT were 89%, 94.6%, and 84%, respectively. This study demonstrates the safety of SRS to push the envelope in treating oligometastatic disease and may negate the need for patients to undergo hippocampal-sparing WBRT protocols aimed at mitigating the adverse effects of WBRT on neurocognition.

#### Emerging Role of the Tumor Microenvironment in Regulating Radiation-Induced Abscopal Effects for Brain Metastases

Perhaps one of the most fascinating yet rare phenomena that is seen following the treatment of a lesion with radiation is the regression of tumors at a distant, non-irradiated site, called the abscopal effect. First described by Mole in 1953 [205], the mechanism of the abscopal effect is not completely understood but likely involves IR-induced modulation of the treated tumor immune microenvironment (TIME), leading to systemic upregulation of the immune system to attack other tumors at distant sites [206]. The biological mechanisms of the abscopal effect are complex and are thought to involve the direct cytotoxic effects of radiation on tumor cells, which reprograms the TME to activate an antitumor immune response leading to the immunogenic cell death of distant site tumors [207]. The recruitment and activation of dendritic cells to the tumor site can lead to the upregulation of cytotoxic T cells, leading to immune priming of the radiated tumor bed [208,209]. Others have reported that the antitumor effect is caused by the shedding of tumor cell debris activating cellular damage-associated molecular patterns (DAMPs) as well as the release of tumor cell antigens that lead to dendritic cell activation and the activation of local lymph nodes to produce tumor antigen specific effector T cells [210,211,212].

A recent systematic review of abscopal effects in patients with brain and spine metastases summarized 16 case reports of 16 patients who had an abscopal effect [213]. Metastatic melanoma and breast cancer brain metastases had the highest reports of abscopal effects. Radiation treatments included WBRT (average dose of 33.6 Gy) and SRS (average dose 21.5 Gy, 1–5 fractions). The average time after delivery of radiation to the detection of the abscopal effect on surveillance imaging was 5.7 months. The most common sites for distant tumor reduction were lung and lymph nodes. Both WBRT and SRS were able to induce an abscopal effect at extracranial sites. Another systematic review of abscopal effects during the treatment of NSCLC or melanoma brain metastases reported synergy between immune checkpoint inhibitors and RT [214]. Studies are underway to determine the effects of dose, fractionation schedule, type of radiation, and sequence of delivery in combination with immunotherapy and targeted therapies to achieve the abscopal effect. Understanding the effects of SRS on the local TIME of primary and secondary brain tumors could lead to novel treatments to further enhance the abscopal effect. Indeed, a seventh “R” of radiobiology—“*reinforcement*”—was recently proposed by Taghizadeh–Hesary to reflect the ability of the TME to protect cancer cells against the effects of radiation therapy [215].

## 5. Radiobiology of Radiosurgery for the Treatment of Arteriovenous Malformations

Radiosurgery to the nidus of arteriovenous malformations (AVMs) leads to luminal occlusion and thrombosis over a course of two to three years. Acute inflammation of the blood vessels leads to release of cytokines that cause vessel hyalinization, luminal wall thickening, myofibroblast proliferation, wall contraction and obliteration, eventually reducing the lifetime risk of hemorrhage to 1% or less. The radiobiological response of AVMs to SRS has been studied extensively and there is ample longitudinal data supporting the safety and efficacy of using SRS to treat these vascular lesions, especially in patients who are not good surgical candidates. Due to the observation that normal vessels rarely decrease in size or occlude after SRS, it is concluded that the abnormal vessels in the AVM nidus have relative sensitivity to IR [5].

Of a large cohort of over 906 patients who had their AVMs treated with Gamma Knife SRS between the years of 1987 and 2004 at the University of Pittsburgh, 145 (16%) had multiple procedures either for reirradiation of an incompletely obliterated nidus or for prospective staging for large AVMs [216]. Of the 602 patients that remained at 24-months follow-up, 74% had total obliteration of their AVMs. Thirty-eight (4%) had an intracranial hemorrhage after treatment during the latency period. Sixteen patients (1.7%) had delayed cyst formation or encephalomalacia as a late radiation effect. Treatment of AVMs with SRS are well-tolerated with few reports of permanent adverse radiation effects [217]. The rich body of clinical outcome data treating AVMs using SRS has allowed us to understand the clinical tolerance of different regions of the brain to radiation, with the most radioresistant in descending order being the frontal, parietal, temporal lobes, and cerebellum, and the most radiosensitive regions being the brainstem, thalamus, and basal ganglia [86,218].

## 6. Radiobiology of Radiosurgery for Trigeminal Neuralgia

SRS has become an effective treatment modality for idiopathic, classical trigeminal neuralgia (TN) and is well-tolerated by most patients. Single-fraction SRS is aimed at a 4 mm isocenter directed to the retrogasserian target, defined at the cisternal portion of the trigeminal nerve at 7–8 mm from the emergence from the front of the pons, visualized using high-resolution T2/FIESTA or CISS MRI using a dose of 90 Gy at the 100% isodose line [219]. Contouring this segment of the cisternal portion of the nerve allows delivery of a higher prescription dose while minimizing the risk of hypoesthesia, which is more commonly seen with radiation exposure to the intrapontine portion of the nerve. Anterior targeting of the trigeminal nerve results in greater long-term pain relief, while posterior targets afford initial relief but may have an increased risk of complications, including dysesthesia, paresthesia, dry eye, deafferentation pain, and keratitis [220]. Pain cessation is reported in 92% of patients at 1 month and 45% achieve long-term pain freedom without medication at 10 years [221].

## 7. Radiobiology of Radiosurgery for the Treatment of Functional Neurological Disorders

Radiosurgery was initially created as a modality to create a lesion for functional neurosurgery. Early experiments in the 1960s demonstrated that high radiosurgical doses > 150 Gy delivered to a small 3 mm × 5 mm diameter area of brain in a goat successfully reproduced a region of focal tissue necrosis within one month [222,223]. Similar findings were seen in rat and human brains at doses up to 200 Gy [224]. Gamma Knife SRS has since been used to treat functional disorders, targeting specific nuclei including the ventro-intermediate nucleus (Vim) of the thalamus for the treatment of essential tremor and the anterior limb of the internal capsule in the case of treatment-refractory obsessive-compulsive disorder (OCD).

### Essential Tremor

SRS to the Vin nucleus of the thalamus has proven to be an effective minimally invasive method to treat patients with intractable tremor [225,226]. Patients are treated with single-fraction SRS at doses ranging from 130 Gy to 150 Gy using a 4 mm shot with GK SRS. A dose delivery above 150 Gy has a greater risk of transient complications, including weakness, contralateral sensitivity, difficulty speaking, and dysphagia. A therapeutic effect is achieved on average by 4.8 months, with a mean complication rate of 17.4%, with higher rates of complications seen in patients who had previously received a radiofrequency thermocoagulation (RFT) pallidotomy. GK thalamotomy has comparable tremor control rates and reduced complication rates compared to deep brain stimulation (DBS) and RFT but has a greater latency of therapeutic effect [225,226].

There is currently a lack of understanding of the radiobiological effects of SRS thalamotomy. To address this, a recent study by Tuleasca and colleagues used modern neuroimaging techniques to study the radiobiology of Vim GK SRS for the treatment of essential tremor as a means of creating a brain connectome. The protocol they established uses multi-omics including structural and functional changes in neuroimaging, genetic analysis, chronobiological information, concomitant therapies, comorbidities, the brain state on pretherapeutic MRI, and tremor phenotype [227]. We look forward to future studies that shed further insight into the radiobiologic effects of SRS for the treatment of multiple types of functional disorders.

## 8. The Future of SRS in the Clinical CNS Space

### Future SRS Applications for the Treatment of Brain Metastases

Several novel trials are currently underway at MD Anderson to try to further define the role of SRS for the treatment of brain metastases. SAFESTEREO (NCT05346367) is attempting to answer whether multifraction SRS can achieve local control in brain metastases of radioresistant histologies between 1.5 and 3 cm in diameter with decreased incidence of radiation necrosis, stratified across two treatment arms (1 or 3 fractions vs. 5 fractions) [228]. A prospective study that is still in the concept phase aims to compare single-fraction SRS to multifraction SRS to treat brain metastases with radioresistant histologies. Another ongoing phase 3 randomized controlled trial, STaRT, compares post-surgical SRT to surgically targeted radiation therapy using Cs-131 collagen tile brachytherapy for the treatment of newly-diagnosed metastatic brain tumors or ROADS (Radiation One and Done Study:NCT04365374).

The use of radiosensitizers to enhance the response of RT across multiple intracranial pathologies could further reduce the need for hypofractionation and also help overcome intrinsic or acquired resistance mechanisms. Work to further characterize and identify synergies between SRS and immune check point inhibitors could help achieve durable intracranial disease control. The sensitization of cells to radiation can be achieved by several approaches, including small-molecule inhibitors or the transgene inhibition of members of the DNA damage repair pathway [229]; hyperthermia to the local tumor milieu [230]; or increasing oxygen delivery to the tumor [231]. The development of inhibitors of the non-homologous end-joining DNA damage repair pathway are of particular interest because of its predominant involvement in the repair of DNA double-strand breaks. Inhibitors of the homologous recombination and base excision repair pathways are also being developed, as well as inhibitors against cell cycle checkpoint proteins [232,233,234,235,236].

There have also been recent preclinical and clinical studies reporting synergy when combining RT with immune checkpoint inhibitors (ICIs) for the treatment of primary and metastatic brain tumors [237,238,239]. This has shown particular promise for the treatment of non-small cell lung cancer and melanoma brain metastases, in which the combination of RT with ICI therapy creates an immunostimulatory effect on the TME [240,241]. This synergistic response is tumor histology specific and checkpoint inhibitor specific and can be tailored to enhance the efficacy of treatment. These combination therapies can, however, increase the rates of radiation necrosis, and treatments to minimize and monitor these adverse neurological events are being used to recognize and treat these potentially adverse outcomes [242,243,244].

Finally, recent advances in robotics and artificial intelligence (AI) have the potential to expand the functionality of robotic-based SRS platforms such as Cyberknife. A recent forum in Palo Atlo, CA, brought together visionary leaders in these two disciplines to discuss the inevitable integration of robots into the open workplace. Dr. Adler’s use of a robotic arm to deliver LINAC SRS (Cyberknife, Accuray) decades ago highlights the power of innovation to improve treatment outcomes for patients. Finally, as we are now firmly rooted in the era of AI, we have used the Google AI search tool to ask the question “How many radiosurgery centers exist worldwide and how many patients have been treated with radiosurgery?” The answer was astounding. Over 800,000 people worldwide have been treated with Gamma Knife, with approximately 120,756 patients with brain tumors treated with SRS since the year 2000. The Stanford Cyberknife program alone has treated over 7000 patients with brain and spine conditions.

In closing, we return to the provocative question posed in the title of our review, “Does the radiobiology of radiosurgery matter?” We recently hosted the inaugural Neuro SRS Course in East Palo Alto, California (https://www.neurosrs.com), bringing together international experts in the field of SRS. At the conclusion of this course, the resounding message was that there is a clear role for understanding the radiobiology of tumors in response to SRS, especially in the modern era of combination precision cancer therapies. Understanding the effects of these novel therapies on synergizing with SRS will allow clinicians to improve the safety profile of these novel treatment regimens, minimize adverse neurological events, and prolong the survival of our patients.

## Figures and Tables

**Table 1:** The R’s of Radiobiology. The principles underlying the biological response of tumor cells to the effects of radiation. Wither’s classic 4 R’s describes the differential effects of dose fractionation on normal tissues compared to tumor tissues. Steel’s 5th R describes the intrinsic radiosensitivity of tumor cells throughout a multi-fractionated treatment schedule. Boustani later introduced the 6th R to account for the response of the tumor immune microenvironment to radiation-induced DNA damage in tumor cells. Recently, Taghizadeh-Hesary proposed the 7th R to stress the reinforcement of the tumor microenvironment in supporting cancer cells against the effects of radiation therapy.

The R’s of Radiobiology in Radiotherapy
Wither’s “Four R’s of Fractionated Radiotherapy”
*Repair* of sublethal cellular damage
*Repopulation* of cells after radiation
*Redistribution* of cells within the cell cycle
*Reoxygenation* of the surviving cells
Steel’s “Fifth R”
*Radiosensitivity* (intrinsic)
Boustani’s “Sixth R”
*Reactivation* of the anti-tumor immune response
Taghizadeh-Hesary’s “Seventh R”
*Reinforcement* by the tumor microenvironment

## Data Availability

Not applicable.
